# Supplementary material for: Prognostic and Predictive Utility of GPD1L in Human Hepatocellular Carcinoma
Source: Int J Mol Sci. 2023 Aug 23;24(17):13113. doi: 10.3390/ijms241713113 (PMC10487989; doi:10.3390/ijms241713113)
Supplement: Supplementary file 1 [file ijms-24-13113-s001.zip › IJMS Submission_Supplementary_Aug22.pdf]

Supplementary: Prognostic and Predictive Utility of GPD1L in Human Hepato-cellular Carcinoma

Table S2. Hazard ratios from Coz proportional hazard model (Overall survival)

	Univariate analysis			Multivariate analysis		
**Characteristic**	**HR[1]**	**95% CI[1]**	**p-value**	**HR[1]**	**95% CI[1]**	**p-value**
Age (years)						
<60	—	—		—	—	
≥60	1.21	0.86, 1.72	0.3	1.43	0.91, 2.25	0.12
Sex						
Female	—	—		—	—	
Male	0.82	0.57, 1.16	0.3	1.00	0.63, 1.60	>0.9
Resection margin status						
R0	—	—		—	—	
R1/R2/RX	2.03	1.22, 3.40	0.007	1.14	0.41, 3.20	0.8
Tumor purity						
<0.75	—	—		—	—	
≥0.75	1.08	0.76, 1.52	0.7	1.22	0.78, 1.89	0.4
Stage						
Stage 1/2	—	—		—	—	
Stage 3/4	2.45	1.69, 3.55	<0.001	1.92	1.21, 3.05	0.006
ECOG Performance Status						
<2	—	—		—	—	
≥2	4.17	2.58, 6.73	<0.001	3.49	2.07, 5.91	<0.001
GPD1L mRNA						
Low	—	—		—	—	
High	1.72	1.21, 2.44	0.002	1.60	1.02, 2.52	0.042

[1]HR = Hazard Ratio, CI = Confidence Interval

Table S3. Hazard ratios from Cox proportional hazard model (Progression-free interval)

	Univariate analysis			Multivariate analysis		
**Characteristic**	**HR[1]**	**95% CI[1]**	**p-value**	**HR[1]**	**95% CI[1]**	**p-value**
Age (years)						
<60	—	—		—	—	
≥60	1.01	0.75, 1.35	>0.9	1.08	0.76, 1.54	0.7
Sex						
Female	—	—		—	—	
Male	0.97	0.71, 1.33	0.9	1.03	0.71, 1.50	0.9
Resection margin status						
R0	—	—		—	—	
R1/R2/RX	1.59	0.99, 2.53	0.053	2.02	1.07, 3.82	0.031
Tumor purity						
<0.75	—	—		—	—	
≥0.75	1.22	0.91, 1.63	0.2	1.42	1.01, 2.01	0.045
Stage						
Stage 1/2	—	—		—	—	
Stage 3/4	2.22	1.60, 3.07	<0.001	2.03	1.38, 3.00	<0.001
ECOG Performance Status						
<2	—	—		—	—	
≥2	3.01	2.00, 4.52	<0.001	2.47	1.57, 3.89	<0.001
GPD1L mRNA						
Low	—	—		—	—	
High	1.35	1.01, 1.82	0.043	1.06	0.74, 1.52	0.7

[1]HR = Hazard Ratio, CI = Confidence Interval

Table S4. TCGA LIHC – GPD1L mTRNA Low vs High Groups

Variable	Overall, N = 359[1]	high, N = 176[1]	low, N = 183[1]	p-value[2]
**TP53**				0.016
Mutant	111 (31%)	65 (37%)	46 (25%)	
WT	248 (69%)	111 (63%)	137 (75%)	
**CTNNB1**				<0.001
Mutant	96 (27%)	30 (17%)	66 (36%)	
WT	263 (73%)	146 (83%)	117 (64%)	
**AXIN1**				0.24
Mutant	23 (6.4%)	14 (8.0%)	9 (4.9%)	
WT	336 (94%)	162 (92%)	174 (95%)	
**ARID1A**				0.91
Mutant	30 (8.4%)	15 (8.5%)	15 (8.2%)	
WT	329 (92%)	161 (91%)	168 (92%)	
**CDKN2A**				0.14
Mutant	11 (3.1%)	3 (1.7%)	8 (4.4%)	
WT	348 (97%)	173 (98%)	175 (96%)	
**ARID2**				0.20
Mutant	20 (5.6%)	7 (4.0%)	13 (7.1%)	
WT	339 (94%)	169 (96%)	170 (93%)	
**RPS6KA3**				0.24
Mutant	14 (3.9%)	9 (5.1%)	5 (2.7%)	
WT	345 (96%)	167 (95%)	178 (97%)	

[1] n (%)

[2] Pearson's Chi-squared test

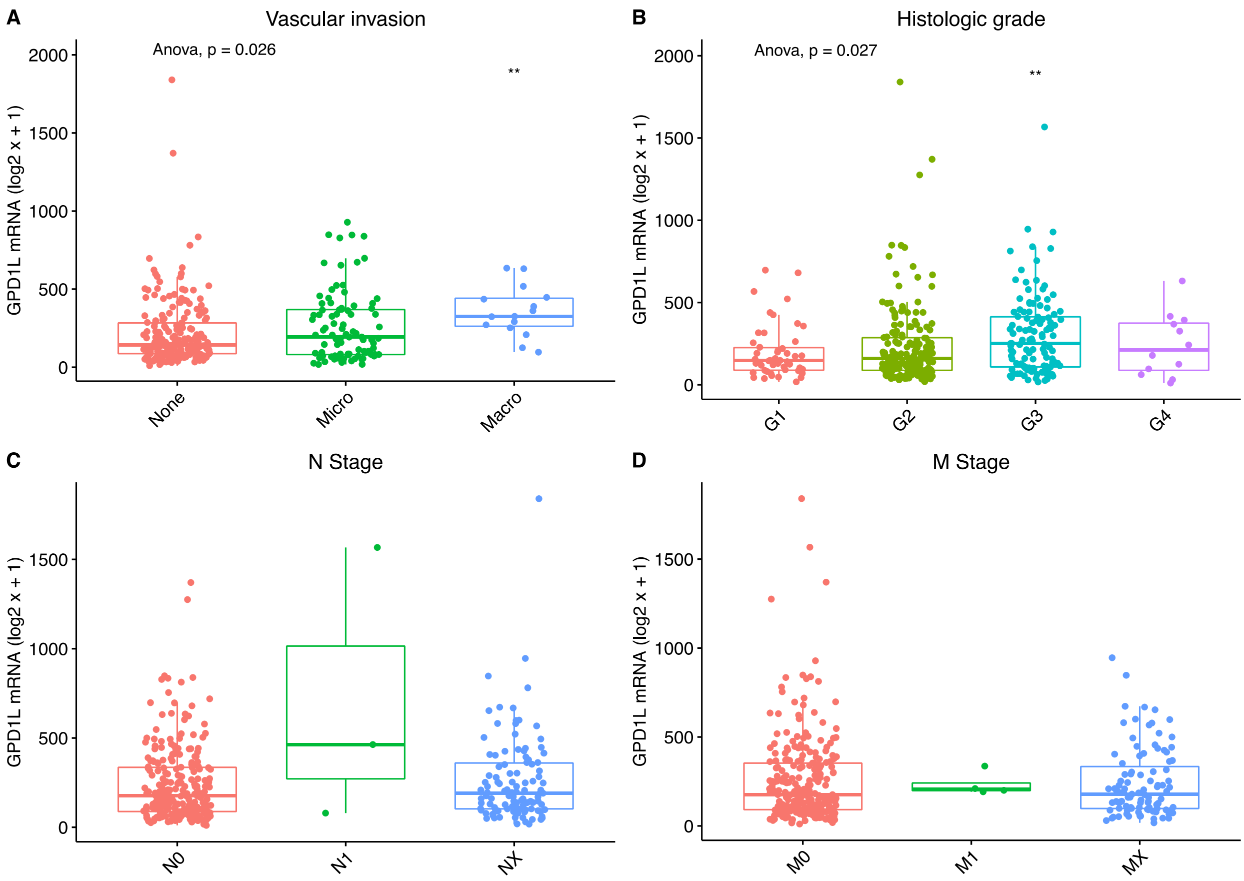

Figure S1. Association of GPD1L mRNA levels with clinicopathological factors (Dataset: TCGA-LIHC). A, Vascular Invasion; B, Histologic Grade; C, N Stage; D, M Stage. Statistical comparisons: Multi-group one-way Anova and 2-sided T-tests, with first category as the reference level (* $P < 0.05$; ** $P < 0.005$) (only significant results shown).

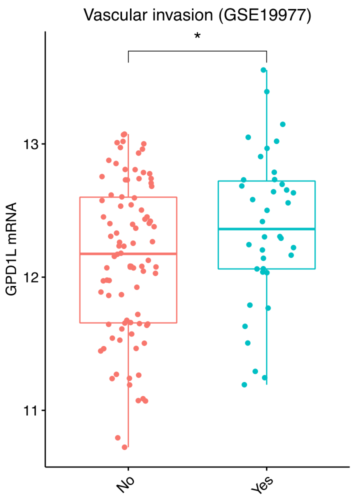

Figure S2. Association of GPD1L mRNA levels with presence or absence of vascular invasion (Dataset: GSE19977). (* $P < 0.05$)

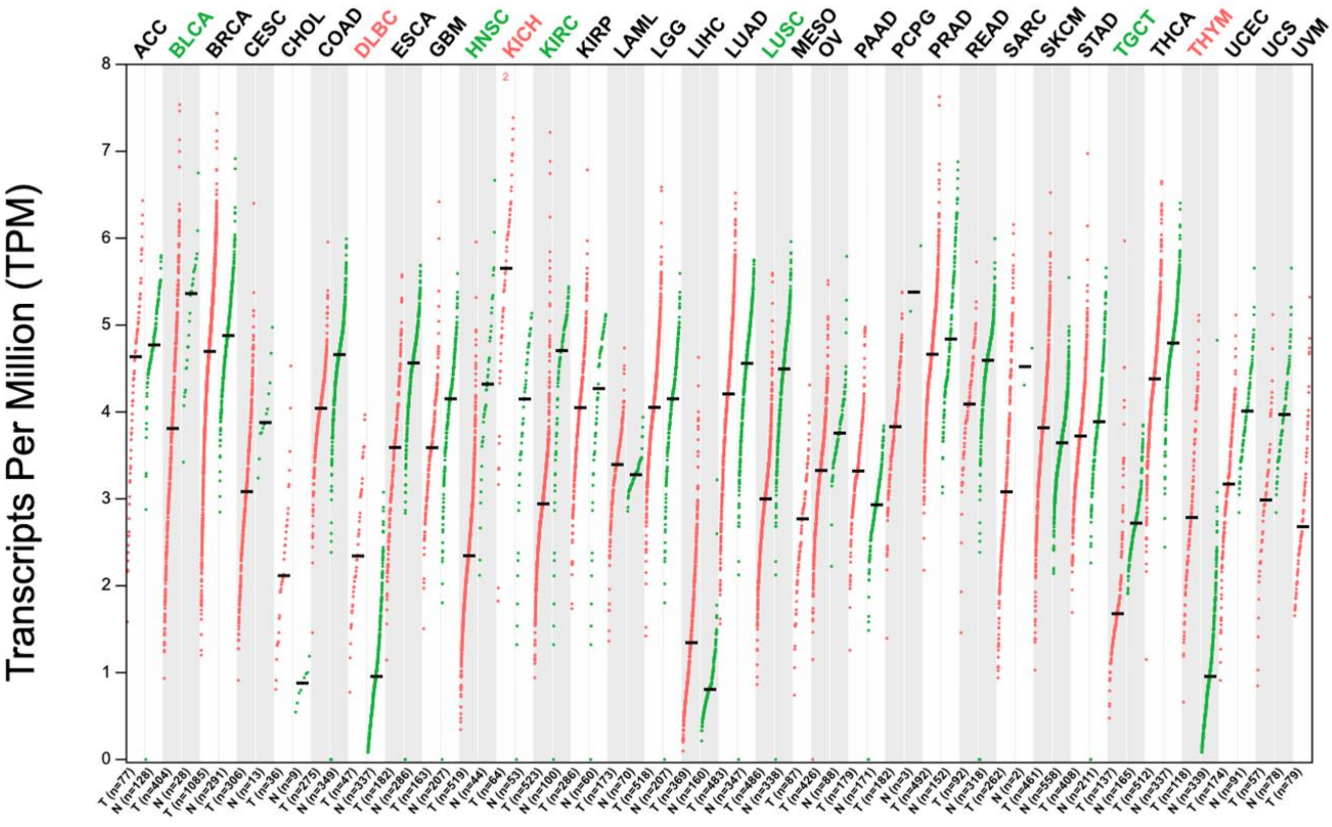

Figure S3. GPD1L mRNA levels in tumour and normal tissue in TCGA

Dataset	P-value	Type	Nums	Mean	STD	IQR
HCCDB1	0.0002359	HCC	100	8.208	1.165	1.516
		Adjacent	97	7.732	0.4690	0.6021
HCCDB3	1.145e−13	HCC	268	1.415	1.066	1.308
		Adjacent	243	0.8859	0.3188	0.3860
		Cirrhotic	40	1.421	0.4091	0.5068
		Healthy	6	0.6495	0.3433	0.6600
HCCDB4	8.809e−45	HCC	240	8.076	0.7790	1.099
		Adjacent	193	7.179	0.2675	0.3449
HCCDB6	3.982e−19	HCC	225	5.295	0.8420	1.214
		Adjacent	220	4.713	0.3447	0.4343
HCCDB7	0.08637	HCC	80	11.73	0.7554	0.7518
		Adjacent	82	11.51	0.8848	1.101
HCCDB11	0.5496	HCC	88	7.446	1.255	1.577
		Adjacent	48	7.582	1.268	1.818
HCCDB12	0.1340	HCC	81	5.109	0.8522	1.124
		Adjacent	80	4.932	0.6243	0.7835
HCCDB13	0.3746	HCC	228	4.197	0.5933	0.5108
		Adjacent	168	4.152	0.3964	0.4350
HCCDB15	0.000007...	HCC	351	7.447	1.264	1.870
		Adjacent	49	6.995	0.4963	0.5600
HCCDB17	0.00001098	HCC	115	9.045	0.8026	1.130
		Adjacent	52	8.656	0.3004	0.4125
HCCDB18	1.674e−13	HCC	212	1.541	0.7475	1.063
		Adjacent	177	1.085	0.3957	0.4500
HCCDB20	0.4469	HCC	53	4.248	0.4562	0.6692
		Adjacent	29	4.322	0.3915	0.4854
		Cirrhotic	8	4.368	0.3300	0.3607
		Healthy	6	3.918	0.1779	0.2697
HCCDB22	5.359e−7	HCC	64	8.848	0.9326	1.299
		Adjacent	30	8.167	0.2435	0.4670
HCCDB24	0.00001102	HCC	115	9.044	0.8030	1.129
		Adjacent	52	8.655	0.3007	0.4102
HCCDB25	1.985e−8	HCC	158	6.337	1.098	1.320
		Adjacent	158	5.716	0.7864	0.9079
HCCDB27	0.01001	HCC	70	7.322	0.8655	1.159
		Adjacent	37	6.995	0.4229	0.5957
HCCDB29	0.04078	HCC	35	8.348	0.7079	1.079
		Adjacent	59	8.074	0.3947	0.5671
		Normal	13	8.200	0.3279	0.4344
HCCDB30	0.004095	HCC	54	1.590	0.6552	1.040
		Adjacent	47	1.265	0.4452	0.5436
		Normal	15	1.114	0.3278	0.3509

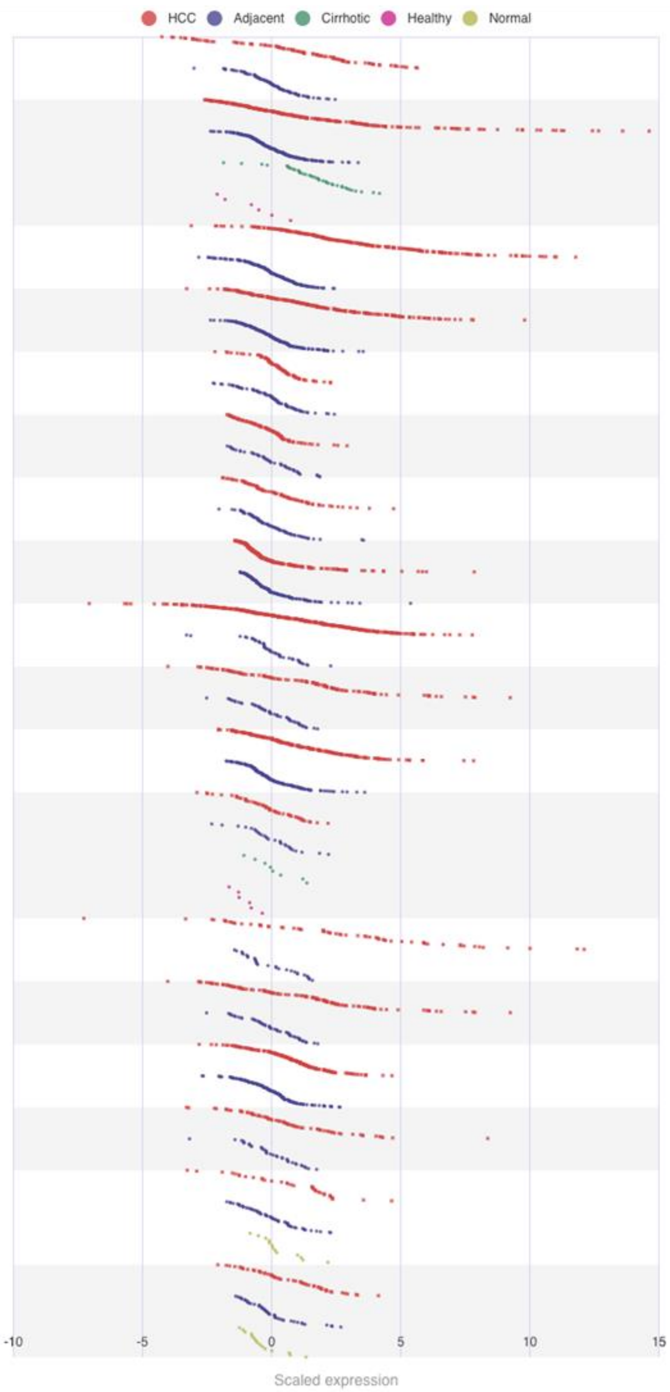

Figure S4. GPD1L mRNA levels in tumour and adjacent normal tissue in HCCDB v2.0 datasets.

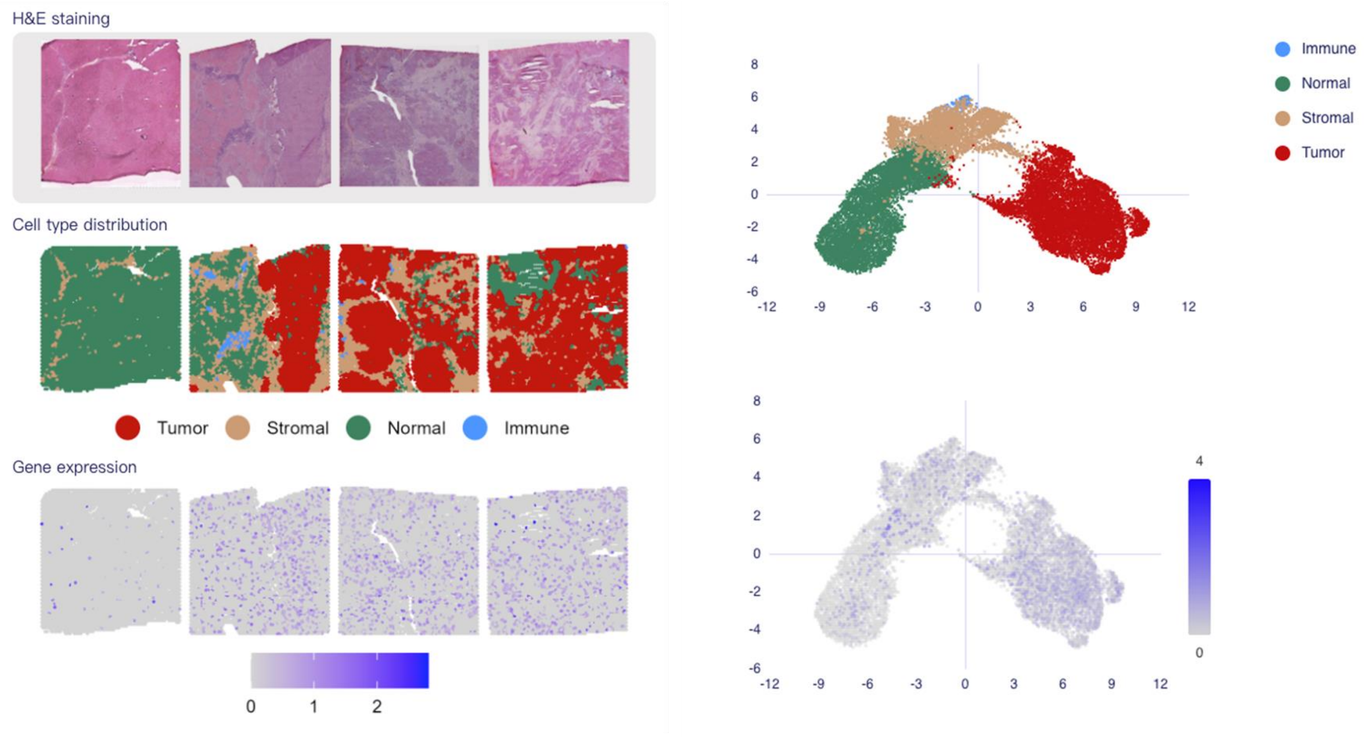

Figure S5. Spatial Transcriptomic HCC-2 dataset from HCCDB v2.0.

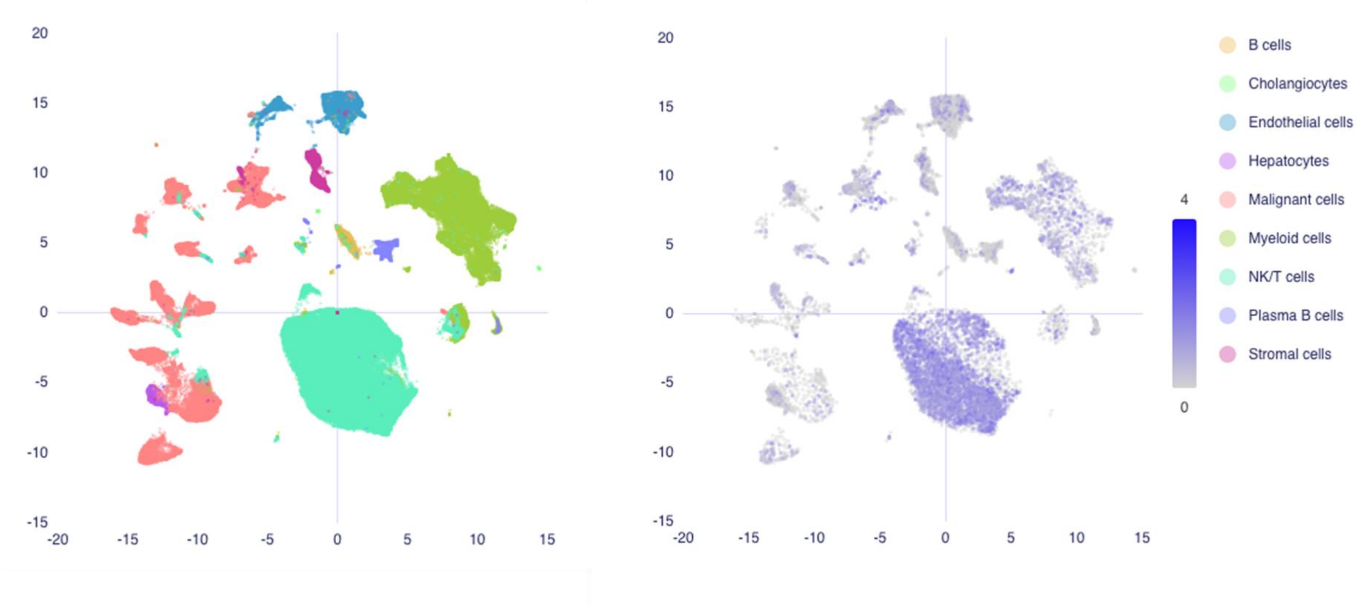

Figure S6. Single Cell RNA-Seq from HCCDB. tSNE plots showing cell population (left) and GPD1L expression in single cells marked in blue (right).

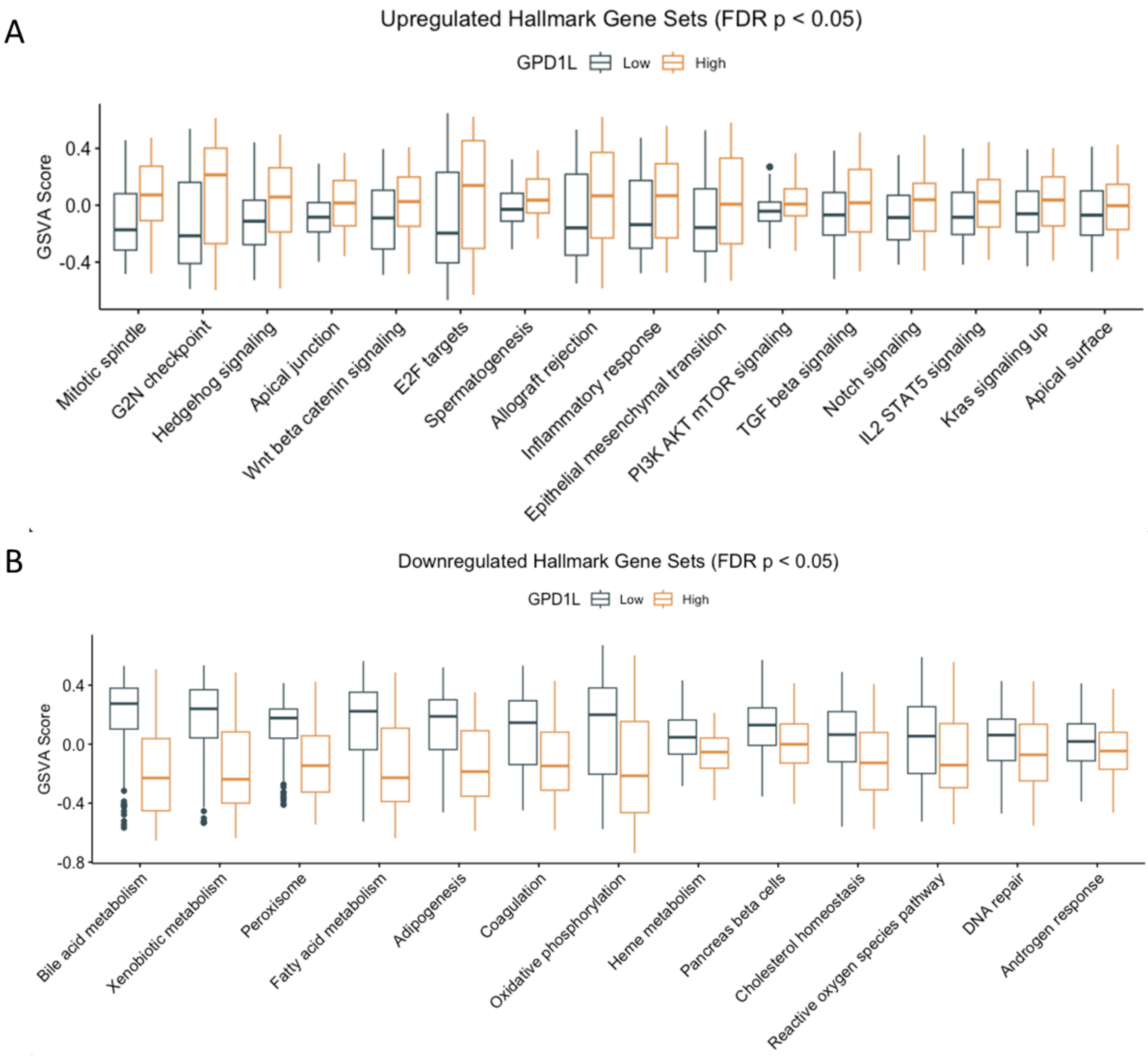

Figure S7. GSVA analysis of HALLMARK gene sets in TCGA-LIHC stratified by GPD1L mRNA levels.

TF		stats	
Term name	Term ID	p_{adj}	$-\log_{10}(p_{adj})$ 0 ≤16
Factor: E2F-3; motif: GGCGGGN; match class: 1	TF:M02089_1	7.577×10^{-18}	
Factor: E2F-1; motif: NNNNGGCGGGAARN; match class...	TF:M09892_1	8.742×10^{-10}	
Factor: E2F-4; motif: NNTTCCCGCCNN; match class: 1	TF:M04823_1	3.629×10^{-7}	
Factor: E2F-6; motif: NGGGCGGGARRNN	TF:M09895	1.350×10^{-6}	
Factor: E2F-4; motif: NGGCGGGAARN; match class: 1	TF:M07084_1	1.706×10^{-4}	
Factor: E2F-1; motif: NKTSSCGC	TF:M00428	8.327×10^{-4}	
Factor: E2F-4; motif: NTTTCSCGCC; match class: 1	TF:M07380_1	1.251×10^{-3}	
Factor: E2F-4; motif: GCGGGAAANA; match class: 1	TF:M02090_1	2.594×10^{-3}	

Figure S8. gProfiler functional enrichment analysis of genes with positive correlation with GPD1L in TCGA-LIHC.

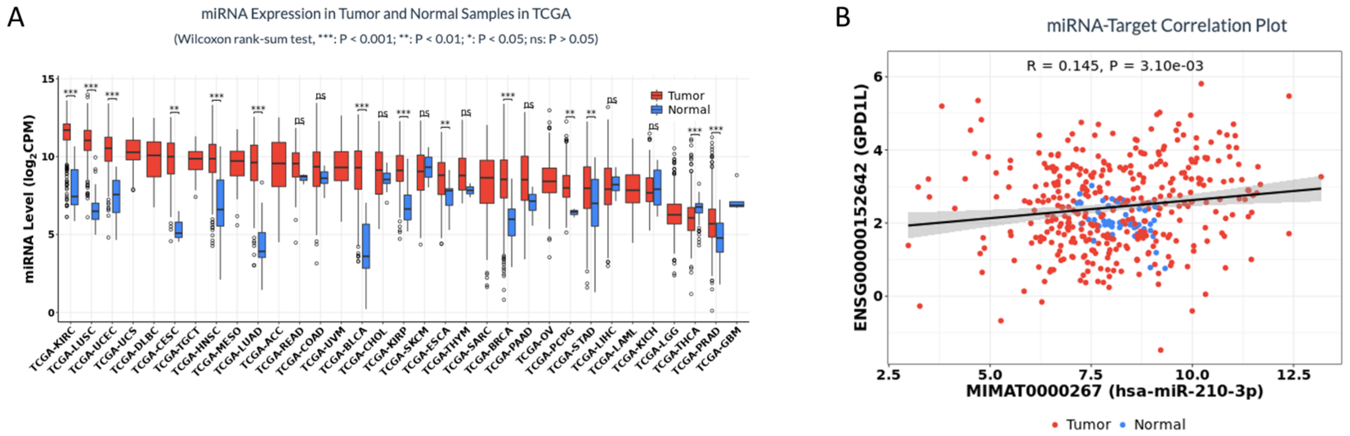

Figure S9. (A) miRNA expression in tumour and normal samples in the TCGA dataset. (B) Correlation plot for miR-210 and GPD1L expression in TCGA-LIHC.

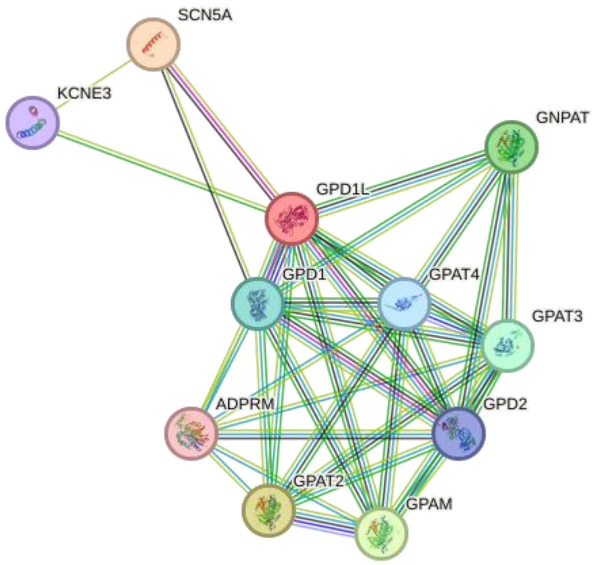

Figure S10. Protein-protein interaction network for GPD1L downloaded from STRING-DB

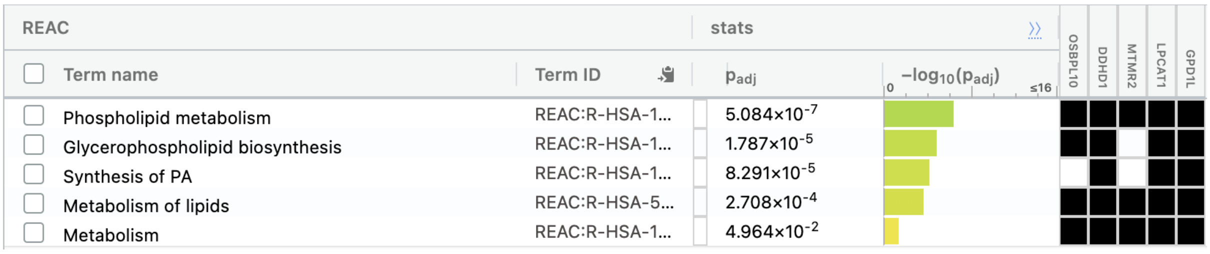

Figure S11. g:Profiler functional annotation of GPD1L co-expressed lipid metabolism genes

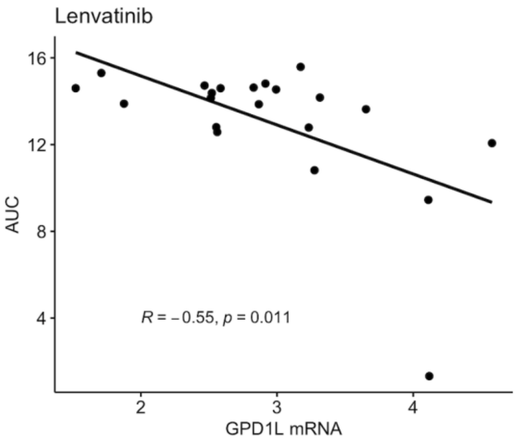

Figure S12. Correlation between hepatocellular cancer cell line mRNA and lenvatinib drug response (area under the curve, AUC). Data source: Rees et al (PMID: 26656090)

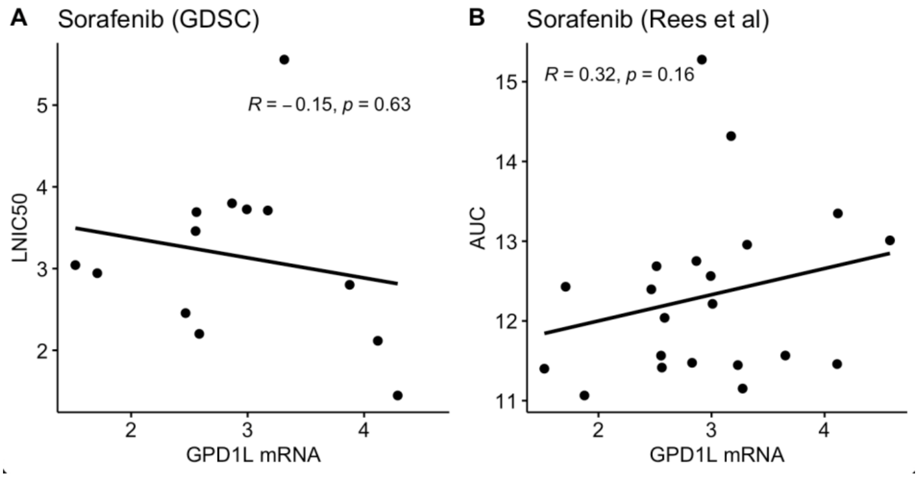

Figure S13. Correlation between hepatocellular cancer cell line mRNA and sorafenib drug response (area under the curve, AUC). Data sources: GDSC and Rees et al (PMID: 26656090)

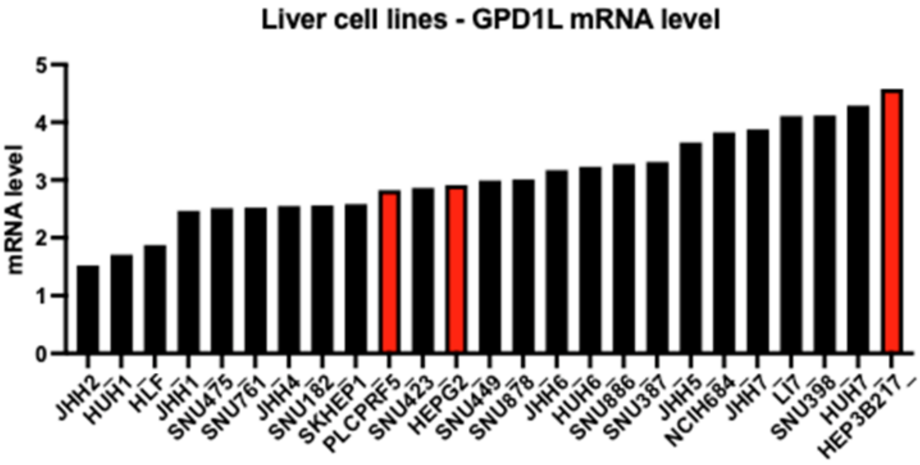

Figure S14. GPD1L mRNA levels in liver cell lines
